# Real-Time Lidar Odometry and Mapping with Loop Closure

**DOI:** 10.3390/s22124373

**Published:** 2022-06-09

**Authors:** Yonghui Liu, Weimin Zhang, Fangxing Li, Zhengqing Zuo, Qiang Huang

**Affiliations:** 1 School of Mechatronical Engineering, Beijing Institute of Technology, Beijing 100081, China; 3120200157@bit.edu.cn (Y.L.); wonk2000@bit.edu.cn (F.L.); 3120215092@bit.edu.cn (Z.Z.); qhuang@bit.edu.cn (Q.H.); 2 Key Laboratory of Biomimetic Robots and Systems, Ministry of Education, Beijing Institute of Technology, Beijing 100081, China; 3 Beijing Advanced Innovation Center for Intelligent Robots and Systems, Beijing 100081, China

**Keywords:** real-time lidar odometry, submap-based loop-closure detection, pose graph optimization, simultaneous localization and mapping (SLAM)

## Abstract

Real-time performance and global consistency are extremely important in Simultaneous Localization and Mapping (SLAM) problems. Classic lidar-based SLAM systems often consist of front-end odometry and back-end pose optimization. However, due to expensive computation, it is often difficult to achieve loop-closure detection without compromising the real-time performance of the odometry. We propose a SLAM system where scan-to-submap-based local lidar odometry and global pose optimization based on submap construction as well as loop-closure detection are designed as separated from each other. In our work, extracted edge and surface feature points are inserted into two consecutive feature submaps and added to the pose graph prepared for loop-closure detection and global pose optimization. In addition, a submap is added to the pose graph for global data association when it is marked as in a finished state. In particular, a method to filter out false loops is proposed to accelerate the construction of constraints in the pose graph. The proposed method is evaluated on public datasets and achieves competitive performance with pose estimation frequency over 15 Hz in local lidar odometry and low drift in global consistency.

## 1. Introduction

Simultaneous Localization and Mapping is a significant issue for mobile robots and autonomous driving vehicles. Vision-based and lidar-based SLAM has been widely studied, proposing a series of noted methods to achieve real-time and high-performance pose estimation. Achieving real-time pose estimation on devices with limited computational resources remains a challenge for both vision and lidar SLAM. For vision-based SLAM using monocular, stereo, or RGB-D cameras, loop-closure detection and relocalization is not a particularly difficult task because a bag-of-words library can be trained in advance, which is a creative approach for data association on a global scale.

Compared with vision-based SLAM, there is a lack of research on loop-closure detection in lidar-based SLAM, although lidar-based methods are more tolerant of illumination and initialization. In our work, we focus on lidar-based real-time pose estimation and a mapping method with loop closure.

A great deal of attention has been paid to lidar-based pose estimation and mapping methods for the last few years. For instance, a feature-based 3D lidar-based SLAM framework called lidar odometry and mapping in real-time (LOAM) [1] achieved both low-drift and low computational complexity. Until now, LOAM and many variants of LOAM have been widely studied because of their state-of-art performance on the public dataset KITTI [2]. Furthermore, usually a Euclidean distance-based loop-closure detection approach is used to minimize the accumulated error as with LeGO-LOAM [3] and LIO-SAM [4].

However, both local data association and the Euclidean-distance-based loop-closure detection method also struggle in a large-scale test if their odometry accumulated error exceeds a certain threshold. Scan matching (also called data association) is one of the most important steps in a SLAM system. The Iterative Closest Points [5] method, which achieves point cloud registration by minimizing the point-to-point Euclidean distance, is the most classic method to obtain a matching result between point clouds.

This is a brute force iterative method to obtain pose transformation between two different point clouds. However, owing to the expensive cost, there are many problems with traditional ICP when applied to specific problems [6]. Therefore, many different versions of the ICP algorithm have been proposed to adapt to different application scenarios, such as PL-ICP [7], NICP [8] and IMLS-ICP [9]. Marchel [10] proposed a modification to the standard method of ICP using three original weighting factors and brought about an improvement in accuracy.

Even so, the ICP method is still not an outstanding method to achieve real-time matching of point clouds when the amount of point cloud data is overly large. The ICP variants mentioned above improve the accuracy, while MIM_SLAM [11] confirms that multi-level ICP (iterative closure point) matching can be used to solve data-association problems.

Additionally, Normal Distribution Transform (NDT) [12] is also a popular point cloud matching algorithm that accelerate the matching process by dividing the grid and fitting the point cloud distribution within the grid using a standard normal distribution. Therefore, the accuracy of the NDT point cloud matching method depends on the size of the grid, and when the grid is fine enough, it will also bring large computational consumption.

In addition to the above methods for raw points matching, point cloud matching based on feature extraction, which was first proposed in LOAM [1], is also a popular method. LOAM has been the level of state-of-the-art pose estimation and mapping method since proposed. Inspired by LOAM, LeGO-LOAM [3] improves the performance of the feature-based pose estimation method by adding steps, such as ground points segmentation.

To improve the performance on a low power embedded computing unit, FLOAM [13], which adopts a non-iterative method when finding the corresponding between point clouds was proposed. Afterward, another variant of LOAM and LeGO-LOAM called LIO-SAM [4] based on an increment optimization iSAM [14] was proposed and reached the level of state-of-the-art lidar-based pose estimation and mapping method. Incremental update of linear matrix and imu pre-integration play important roles in the LIO-SAM method.

Similarly, many methods, such as LIO-Mapping [15], MILIOM [16] and Fast-lio [17] integrate imu preintegration information into point cloud matching, instead of using imu to remove point distortion. Moreover, a fast LiDAR-Inertial-Visual odometry called Fast-Livo [18], which fuses vision with traditional lidar odometry was proposed, achieving real-time performance at the level of state-of-the-art. Different from traditional feature extraction methods, a novel method of scan matching based on Fast Fourier Transform(FFT) where the point clouds data are converted to images and FFT is used for pose estimation between images was proposed by Jiang [19].

The pose estimation and mapping method by both raw points matching and feature points matching will lead to the accumulation of errors. Therefore, loop detection and pose optimization are crucial. Cartographer [20] used branch-and-bound accelerated matching for loop-closure detection. ORB-SLAM [21] used a bag-of-words library for loop-closure detection. Algorithms, such as LCD [22], use neural network for loop-closure detection.

Furthermore, a novel method called Scan Context [23], which uses the global descriptor extracted by scan context for loop-closure detection was proposed. Furthermore, Scan Context was proven to be effective in LeGO-LOAM-SC [24]. Loop detection often means positioning in complex environments. Marchel [25] proposed a location method based on fixed position beacons and the EKF optimization method, which has been applied in port approach fairways. There are two popular approaches for handling the remaining error accumulation, called filter-based and graph-based methods [20,26].

Generally speaking, the typical filter-based SLAM is mostly 2D lidar SLAM designed for indoor environments, cannot be used to optimize the loop and proved to be difficult to apply to large-scale outdoor scenes. For instance, the famous Gmapping [26] algorithm works well for small-scale indoor mapping; however, it struggles when applied to large scenes. It is clear that the graph-based method is the mainstream approach for dealing with accumulated error. Since graph-based SLAM was first proposed in [27], quite a few excellent graph optimization methods have been proposed.

One of the most popular graph-based approaches is Bundle Adjustment [28], which uses nodes to represent poses as well as landmarks and edges to represent constraints generated from observations. Many vision-based SLAM frameworks, such as ORB-SLAM [21] and VINS-MONO [29] utilize Bundle Adjustment to optimize the pose of the camera and landmarks. In lidar-based SLAM, pose graph that does not optimize the pose of landmarks is widely used. In order to solve the pose graph optimization problem, the most common method is to solve a large non-linear least-squares optimization problem.

However, until the famous Sparse Pose Adjustment [30] that uses sparse linear methods to reduce time complexity was proposed, it appeared to be impossible to use pose graph optimization on a SLAM problem because optimizing the pose graph was too time-consuming.

In this paper, we propose a graph-based lidar SLAM system consisting of real-time local lidar odometry where feature submaps are constructed to describe the local environment and pose graph optimization after submap-based loop-closure detection to tackle the problems mentioned above. We consider that the error in the same submap with several consecutive scans insertion is small enough to be ignored. The two adjacent edge submaps and two surface submaps maintained at the same time ensure the continuity of the local odometry.

A non-iteration scan-to-map matching is used to estimate the optimal pose of the scan in the submap and then edge points and surface points are inserted into edge submaps and surface submaps, respectively. That is to say, we do not use the Iterative Closest Points (ICP) Algorithm, a brute force matching method, for local data association. The rich local information in the submap and the use of 3D KD-tree accelerate the local data association. In this way, the local lidar odometry can achieve a performance of more than 15 Hz on a platform with limited computing resources.

To maintain the size of the submap, the finished feature submap that is inserted with feature points over a certain number of times will be frozen and added to the pose graph prepared for the global data association running in the background. Therefore, computationally expensive loop-closure detection is separated from the local lidar odometry module. Compared with other existing methods, we perform better on the real-time performance of local lidar odometry and the accuracy of global localization. The main contributions of our work can be summarized as follows:

1. A graph-based lidar SLAM with local lidar odometry and loop-closure detection separated from each other, which achieves local high accuracy and global low drift.
2. An efficient feature submap construction and update method using nonlinear least-squares based non-iteration scan-to-map matching.
3. A novel method for solving the initial value of loop detection optimization problem and a global data association method based on feature submap, which is used to describe the local environment.

The rest of this paper is outlined as follows. Section 2 presents the system overview of the proposed SLAM system and describes the details of the proposed SLAM system including local lidar odometry, loop closure and pose graph optimization. Section 3 shows the experiment results. Finally, we highlight some conclusions and introduce our future work.

## 2. Materials and Methods

**Figure 1:**
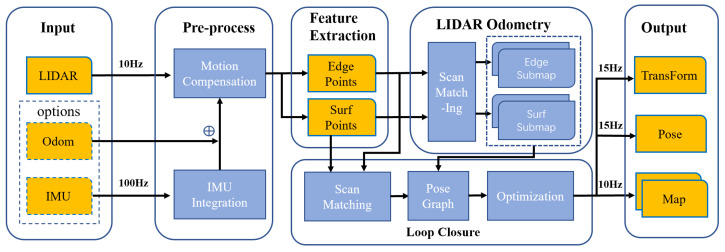
A system overview of the proposed SLAM framework. The main part of this method consists of feature extraction, local lidar odometry and loop closure. Without considering the limitation that the maximum frequency of the input point cloud is 10 Hz, we can achieve a 15 Hz pose estimation and 10 Hz mapping output.

The architecture of our proposed feature-based SLAM framework is shown in Figure 1. Our system consists of front-end local lidar odometry and back-end loop closure and pose optimization. In the beginning, feature extraction is performed first, using the method similar to LOAM [1]. Edge and surface points are extracted by evaluating the smoothness of the local area.

After the feature extraction process, edge points and surface points are inserted into the edge submap and surface submap at the best-estimated position, respectively, which is considered accurate enough for a lidar scanning period. To avoid unnecessary errors and ensure that there are always enough point clouds in the active submap during scan matching, we designed that half of the regions of two adjacent submaps, edge submap or surface submap, are composed of the same lidar points.

In order to reduce the cumulative error, we placed both the extracted feature points and finished feature submaps in the pose graph, processing with loop detection and pose optimization regularly. Once a submap is finished, there will not be new lidar points inserted into feature submaps. In our loop-closure-detection module, while extracted feature points are inserted into submaps, a scan matching process is running independently in the background to judge whether the scan of feature points stored in the pose graph matches any feature submap at a finished state successfully. If a successful scan match between feature points and a feature submap is found, a loop closure constraint will be added to an optimization problem.

In order to solve the optimization problem, we extend the well-known Sparse Pose Adjustment (SPA) [30] to 3D poses and then minimize the cumulative error on the local lidar odometry. In the end, local lidar odometry and loop-closure detection operate independently of each other, and thus there will be no loss in the real-time performance of local lidar odometry despite loop closure’s time-consumption.

### 2.1. Local Lidar Odometry

Local lidar odometry optimizes the pose, x=(t,q) consisting of a translation t=(x,y,z) and a rotation quaternion q=(w,x,y,z), when inserting feature points to submaps, respectively. Generally, the pose *x* also can be represented as a transformation matrix *T*. This optimizing process is called scan matching in this paper. To correct the distortion of mechanical 3D lidar, a constant angular velocity and linear velocity model is used to predict the relative transformation of the point cloud with respect to the scanning start time.

There is no doubt that local lidar odometry calculated by scan-to-map scan matching will accumulate errors with the increase in the number of nodes and submaps. Pose graph optimization will be used to minimize this kind of error in the subsystem loop closure, which will be introduced in Section 2.2.

#### 2.1.1. Feature Extraction

As is known to us all, a frame of lidar contains a huge amount of data, making the scan matching process difficult to complete in real-time. In order to improve the efficiency of scan matching and ensure the real-time performance of odometry, we decided to extract feature points. Unlike image information, the resolution of lidar points in horizontal and vertical directions is different. Points in the vertical direction are more sparse than those in the horizontal direction, and thus we have to extract feature points according to the distribution of points in the horizontal direction. Therefore, we extract edge points and surface points according to the curvature in horizontal direction of the local region calculated by:

(1) $$C=\frac{1}{\left|S||\right|p_{i}\left|\right|}\sum_{j\epsilon S,j\neq i}\left|\right|\left(p_{i}-p_{j}\right)\left|\right|$$

where S represents the adjacent area of $p_{i}$ when we calculate the curvature of point $p_{i}$, and |S| represents the number of points in this area.

If the value of C at point $p_{i}$ exceeds a threshold $c_{E}$, then it will be classified as edge points, and if less than a threshold $c_{s}$, it will be classified as surface points.

#### 2.1.2. Scan Matching

In the beginning, we take the initial pose $x_{0}=\left(t_{0},q_{0}\right)$ of the lidar frame as the pose of the first local submap frame and global map frame. $t_{0}$ and $q_{0}$ are set to (0,0,0) and (1,0,0,0), respectively. Submap construction is equivalent to a repeatedly scan-to-map scan matching process because with the lidar frame pose $x_{i}$, denoted as $T_{i}$ in the local lidar odometry frame, feature points extracted from a 3D lidar raw data can be transformed into the submap frame and then inserted into an edge submap or surface submap, as calculated by:

(2) $$p_{submap}=\left[\begin{array}{cc} R_{i} & t_{i}\\ 0 & 1 \end{array}\right]\left[\begin{array}{c} p\\ 1 \end{array}\right]$$

where p=(x,y,z) is a feature point, $R_{i}$ is rotation matrix of $T_{i}$, $t_{i}$ is the translation vector of $T_{i}$.

We regard scan matching as a nonlinear optimization problem that minimizes the error of point cloud registration to find the optimal pose. Therefore, we need to find several nearest edge points in the submap for current edge points and surface points in the submap for current surface points. Similar to LOAM, we use a 3D KD-tree to speed up the k-nearest neighbor search. With two kinds of extracted feature points, the distance error calculated from the edge feature and surface feature is added to the optimization problem, respectively.

The distance $d_{E}$ from point to line between current edge point $p_{E}$, and its corresponding edge points in the submap can be calculated by:

(3) $$d_{E}=\left|\left(Tp_{E}-p_{E}^{Map}\right)\times n_{E}^{Map}\right|$$

where $p_{E}^{Map}$ is the center point of the found nearest corresponding points in the edge submap and $n_{E}^{Map}$ is the unit direction vector of the found nearest corresponding points in the edge submap if they are verified to be in the same line.

The distance $d_{s}$ from point to plane between the current surface point $p_{s}$ and its corresponding surface points in the submap can be calculated by:

(4) $$d_{s}=\left(Tp_{s}-p_{s}^{Map}\right)\cdot n_{s}^{Map}$$

where $p_{s}^{Map}$ is the center point of the found nearest corresponding points in the surface submap and $n_{s}^{Map}$ is the unit norm vector of the found nearest corresponding points in the surface submap if they are verified to be in the same plane.

The transformation *T* consisting of a rotation and a translation that transforms current feature points to edge submap, and the surface submap can be calculated by solving the nonlinear optimization problem using the Gauss–Newton method to minimize:

(5) $$T^{*}=\arg\min_{T}\sum_{p_{E}\epsilon P_{E},p_{s}\epsilon P_{s}}\left(d_{E}+d_{s}\right)$$

where $P_{E}$ and $P_{s}$ are the sets of current feature points.

For Equation (5), we can obtain the optimal pose by solving the non-linear problem. The Jacobian matrix of $d_{E}$ residual can be calculated by:

(6) $$\begin{array}{cc} J_{E} & =\frac{\partial d_{E}}{\partial (Tp_{E})}\frac{\partial Tp_{E}}{\partial T}\\ \frac{\partial Tp_{E}}{\partial T} & =\left[\begin{array}{cc} -{\left(Tp_{E}\right)}^{\wedge } & I \end{array}\right]\\ \frac{\partial d_{E}}{\partial (Tp_{E})} & =n_{⊥}\cdot {\left(n_{E}^{Map}\right)}^{\wedge }\cdot \left[\begin{array}{cc} -{\left(Tp_{E}\right)}^{\wedge } & I \end{array}\right]\\ n_{⊥} & =\frac{\left(Tp_{E}-p_{E}^{Map}\right)\times n_{E}^{Map}}{d_{E}} \end{array}$$

Furthermore, we can also calculate the Jacobian matrix of the $d_{H}$ residual by:

(7) $$J_{s}=n_{s}^{Map}\cdot \left[\begin{array}{cc} -{\left(Tp_{s}\right)}^{\wedge } & I \end{array}\right]$$

∧ in Equations (6) and (7) represents the cross-product matrix of the acted vector, and *I* stands for the identity matrix.

When the non-linear optimization problem converges, the current pose is updated to its optimal solution. At the same time, the feature points are transformed into the submap frame by the updated pose and inserted into feature submaps, while the updated pose and corresponding feature points are added to the pose graph, preparing for the loop-closure detection running in the background. If a submap is inserted more than a certain number of times, it will be marked as finished state, and a new submap will be created for the following scan matching process. Significantly, the edge submap and surface submap will always be updated at the same time.

### 2.2. Loop Closure and Pose Optimization

Concerning a SLAM problem, what we expect to do is to achieve high accuracy for a few consecutive scans with no distinct drift on the global scene. In general, loop detection is equivalent to global localization and pose recovery. After computing the loop-closure detection constraints and adding them to the pose graph, we extend the famous Sparse Pose Adjustment [30] to 3D poses to optimize the set of poses and constraints in the back-end of our SLAM system.

While a scan from lidar is inserted into the submap at the front-end, which is also called real-time lidar odometry, a scan matcher for finding the corresponding relative pose between nodes and submaps stored in memory is run in the background to determine whether there is a loop closure. Our approach to loop-closure detection and pose optimization are presented in this section.

#### 2.2.1. Error Formulation

To construct the residual equation of the pose optimization problem, we use $X_{i}$ and $M_{i}$ to represent the node and submap at the global coordinate system, while $h_{ij}$ is used to represent the constraints directly measured between node $X_{i}$ and node $X_{j}$. At the same time, the offset between node $X_{i}$ and node $X_{j}$ is measured in $X_{i}$’s frame with precision matrix $\Lambda_{ij}$ in denoted as $z_{ij}$. Moreover, the process of calculating $z_{ij}$ meaning loop-closure detection and constraint construction is discussed in Section 2.2.2. The constraint h(i,j)) between node $X_{i}$ and $X_{j}$ can be calculated by:

(8) $$\begin{array}{cc} h(i,j) & =T_{X_{i}}^{-1}T_{X_{j}}\\ \; & =\left[\begin{array}{cc} R_{i}^{-1}R_{j} & R_{i}^{-1}\left(t_{j}-t_{i}\right)\\ 0 & 1 \end{array}\right] \end{array}$$

where $T_{X_{i}}$ is the pose of node $X_{i}$ and $T_{X_{j}}$ is the pose of node $X_{j}$.

With the constraint h(i,j), the error function at each loop closure and the total pose optimization error of the system can be described as:

(9) $$\begin{array}{cc} e_{ij} & =z_{ij}-h\left(i,j\right)\\ E^{2} & =\sum_{ij}e_{ij}^{T}\Lambda_{ij}e_{ij} \end{array}$$

#### 2.2.2. Loop-Closure Detection and Constraint Construction

**Figure 2:**
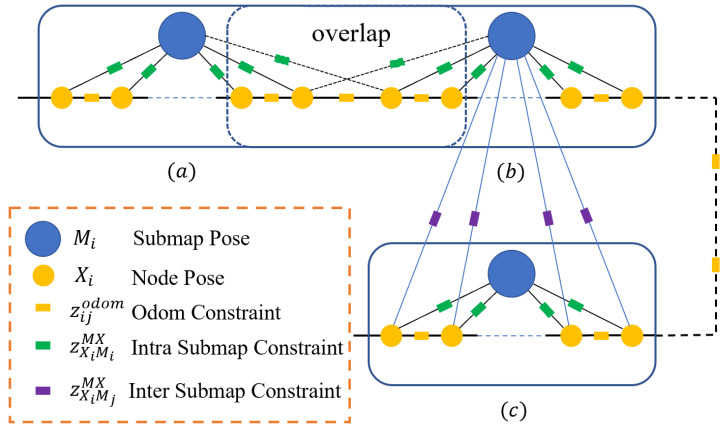
The system structure of constraint construction. (**a**,**b**) Two adjacent submaps, which are contacted by adjacent submap constraints, equivalent to odometry constraints actually. (**c**) A submap that triggers successful loop-closure detection on odometry, nodes of which can be matched with (**b**). The loop-closure constraint is called inter submap constraint in this system. In addition, inter submap constraint represents the transformation between the submap pose and node pose. The odom constraint represents the constraint between adjacent nodes on the odometry.

Scan matching is the most crucial part of loop-closure detection. In analogy to the well-known algorithm Cartographer, loop-closure detection means matching scans and submaps in a force iterative way, to ensure that the possible loop closure constraints are not missed. One primary problem with this method is that a lot of computing resources are wasted on invalid scan matching. In this section, we focus on solving this problem. An overview of the constraint construction module is shown in Figure 2.

As described in Section 2.1.2, scan matching is solving nonlinear optimization problems; therefore, a successful and accurate scan matching relies heavily on the initial optimization value. In general, we can use the NDT [12] matching method to obtain the initial value of a scan-matching optimization problem. High-precision lidar odometry can provide a very good optimized initial value for loop detection. Therefore, if the node and submap are too far apart on the odometry, we will consider that there is no loop closure constraint between them. With high-precision odometry, we can obtain a more accurate initial value by the following methods.

**Figure 3:**
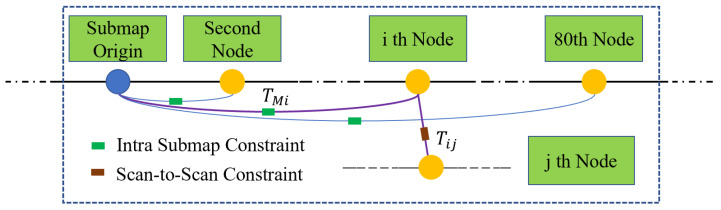
The process of solving node to submap constraints. When node *j* comes to a position not too far from node *i*, we find the transformation from node *i* to node *j* using ICP brute force matching.

When we compute the possible constraints on node $X_{i}$ and submap $M_{j}$, the node closest to $X_{i}$ in the submap $M_{j}$ is found as the distance threshold to filter impossible matches. As is shown in Figure 3, $T_{ij}$ is obtained by scan matching between $X_{i}$ and the nearest node in submap $M_{j}$, using the method of ICP. In this way, we obtain an accurate initial value of nonlinear optimization by:

(10) $$T_{0}=T_{Mi}*T_{ij}$$

The distance-based initial value calculation will be performed on the new inferred odometry after optimization. Then, the next process of scan matching is exactly the same as that mentioned in Section 2.2.2.

#### 2.2.3. Linear System

The optimal global node poses and submap poses are found by minimizing the total error $E^{2}$ in the least-squares Equation (9).

The Levenberg–Marquardt (LM) linear system is:

(11) $$\left(H+\lambda diagH\right)\Delta x=J^{T}\Lambda e$$

where λ is a small positive multiplier, and Λ, *J* and *H* are defined as:

(12) $$\begin{array}{cc} \Lambda & =\left[\begin{array}{ccc} \Lambda_{11} & \; & \\ \; & \ddots & \\ \; & \; & \Lambda_{mn} \end{array}\right]\\ J & =\frac{\partial e}{\partial x}\\ H & =J^{T}\Lambda J \end{array}$$

We can also use the quaternion q to represent the same rotation as the rotation matrix R. With this premise, the Jacobian matrix in Equation (11) can be calculated by:

(13) $$\begin{array}{cc} \; & \frac{\partial e_{ij}^{t}}{\partial t_{i}}\equiv -R_{i}^{T}\;\;\;\frac{\partial e_{ij}^{t}}{\partial t_{j}}\equiv R_{i}^{T}\\ \; & \frac{\partial e_{ij}^{t}}{\partial q_{i}}\equiv {\left[\begin{array}{c} R_{i}^{T}\left(t_{j}-t_{i}\right) \end{array}\right]}_{X}\;\;\;\frac{\partial e_{ij}^{t}}{\partial q_{j}}\equiv 0\\ \; & \frac{\partial e_{ij}^{q}}{\partial q_{i}}\equiv -[0\;I]{\left[q_{z}⊗q_{j}^{*}⊗q_{i}\right]}_{L}\left[\begin{array}{c} 0\\ \frac{1}{2}I \end{array}\right]\\ \; & \frac{\partial e_{ij}^{q}}{\partial q_{j}}\equiv -[0\;I]{\left[q_{z}^{*}⊗q_{j}^{*}⊗q_{i}\right]}_{L}\left[\begin{array}{c} 0\\ \frac{1}{2}I \end{array}\right] \end{array}$$

where $q_{z}$ represents the rotation of loop closure constraint $z_{ij}$.

After linearizing the optimization problem, the extended Sparse Pose Adjustment method is used to solve the special linear overdetermined equation where *H* is a large sparse matrix. Then, we obtain a global optimal solution of node pose and submap pose after loop closure detection.

## 3. Results

**Figure 4:**
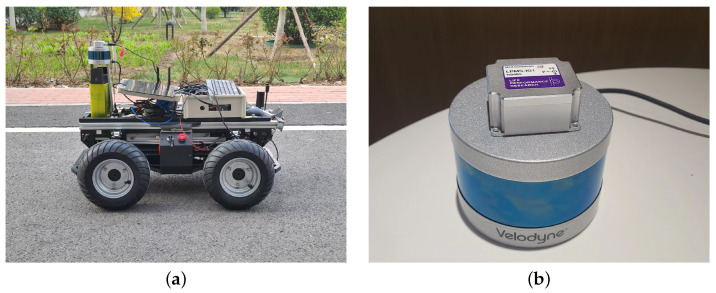
The experiment platform. Our dataset is collected on automated guided vehicle (**a**) using sensor component (**b**).

In order to evaluate the proposed method, we conducted a series of experiments on our mobile platforms, an automated guided vehicle, in outdoor environment and public datasets. The sensor module attached to our mobile consisted of a Velodyne-VLP16 lidar and an LPMS-IG1 IMU. The platform and sensor module are shown in Figure 4. A real scene dataset was collected from campus using the automated guided vehicle platform. To compare the proposed method with other approaches, such as LOAM and FLOAM, we evaluated the proposed method on the well-known public dataset KITTI [2]. All the experiments, including the public dataset and the datasets collected by our vehicle, were executed on a laptop with an Intel i7-6700HQ at 2.60 GHz, a computing platform with relatively limited computational resources.

### 3.1. Experiment on Our Platform

The experiment was conducted to evaluate the performance of our algorithm when applied to robots working in an outdoor environment. In this experiment, an autonomous guided vehicle that was used to verify the performance of our proposed approach was designed to navigate autonomously on a scene that was close to 180 m × 180 m on the campus.

**Figure 5:**
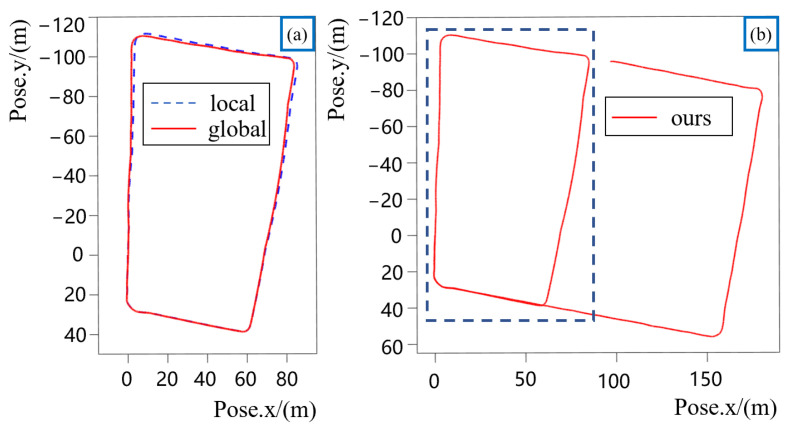
Trajectory results on our dataset. Trajectory (**a**) shows the comparison of the trajectory with loop closure constraint and the trajectory without loop closure constraint of the road segment designed with loop closure. Trajectory (**b**) shows the complete trajectory.

**Figure 6:**
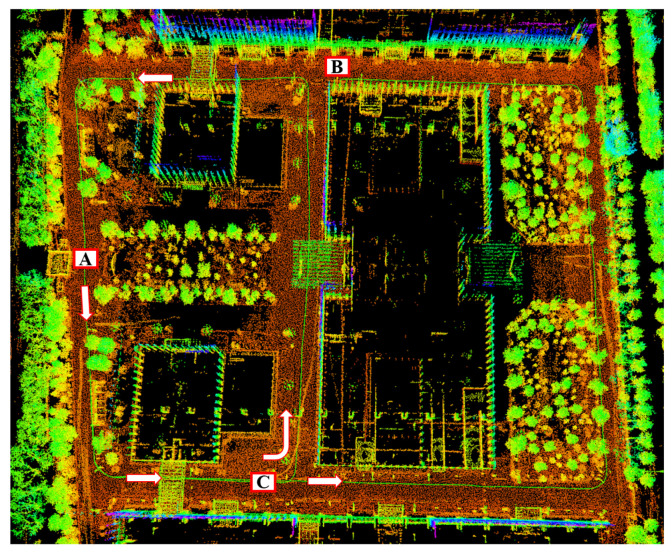
Mapping results on our own dataset using our proposed method. The capital letter A,B,C are used to indicate the road segment information.

The trajectory obtained using our approach and the final map conducted using our approach are shown in Figure 5 and Figure 6. In Figure 6, we show the motion path for collecting the data and mark the direction of movement of our vehicle, starting from A and ending at B. We also design the road segments A–C with loop closures so that the vehicle passes by A–C twice. Combined with the trajectory and map conducted using our approach, we achieve global consistency and a good mapping result. However, due to the poor reliability of RTK in the environment of high buildings, we did not conduct an error evaluation on the trajectory for the time being.

Intuitively, because the real path when we collect the dataset coincides at the loop closure segment, segments A–C in Figure 6, the start-to-end translation error when the vehicle comes to A for the second time can reflect the robustness of the proposed approach. The experimental results show that the start-to-end error is less than 1.3 m when processing more than 6000 frames of lidar data.

### 3.2. Experiment on the KITTI Dataset

**Figure 7:**
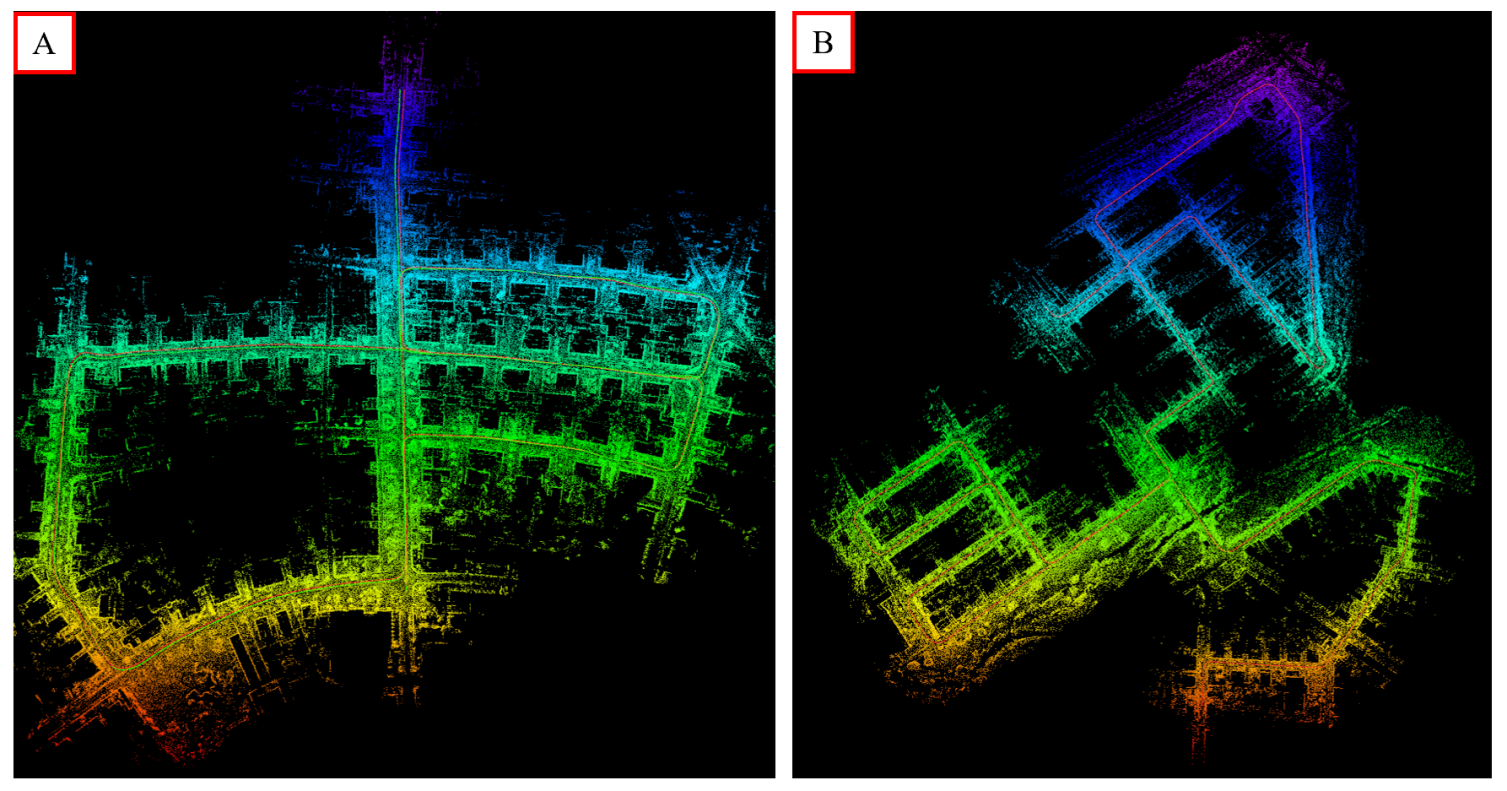
Examples of our approach on the public dataset KITTI. (**A**,**B**) represent the mapping results on sequence 05 and sequence 08, respectively.

KITTI equipped with cameras, lidar, IMU and GPS and is one of the most popular public datasets to evaluate the performance of pose estimation and mapping approaches. To show the mapping performance of our proposed method, the point cloud maps comprising edge and surface feature points built on KITTI sequence 05 and sequence 08 are presented in Figure 7.

**Figure 8:**
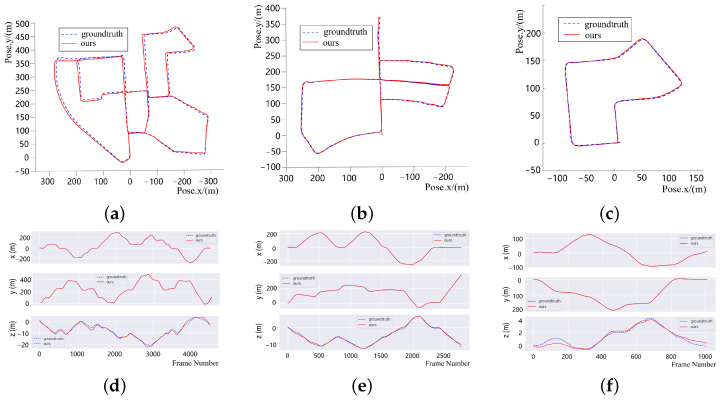
The experimental results on KITTI. The trajectory output of our proposed approach is plotted as a red solid line, while the ground truth is plotted as a blue dashed line. (**a**) KITTI00 trajectory; (**b**) KITTI05 trajectory; (**c**) KITTI07 trajectory; (**d**) KITTI00 error distribution; (**e**) KITTI05 error distribution; and (**f**) KITTI07 error distribution.

The evaluation experiment is mainly conducted on KITTI dataset sequences 00, 05 and 07. The comparison between the trajectory obtained using our proposed method and the groundtruth is shown in Figure 8.

We used the open-source Python package Evo developed for handling and comparing the trajectory output of SLAM algorithms to evaluate our algorithm. As shown in the error distribution figures in Figure 8, the conclusion that an unbiased estimation of the positioning is achieved can be obtained. As of the addition of loop closure, we performed better LOAM and FLOAM on teh global consistency from the overall trajectory.

**Figure 9:**
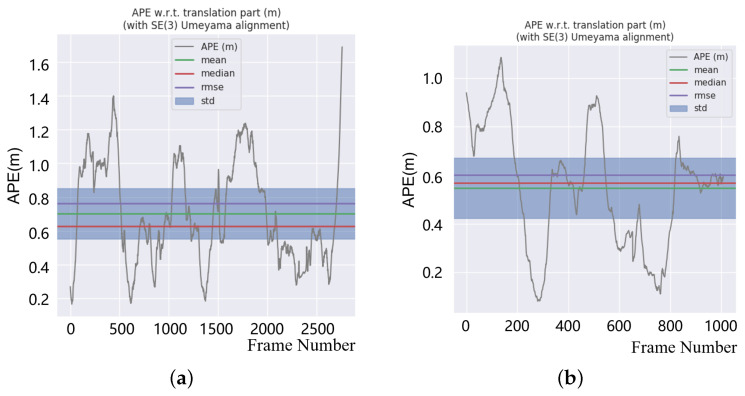
APE result on KITTI sequence 05 and sequence 07. (**a**) KITTI05 APE result and (**b**) KITTI07 APE result.

Then, we conducted a further error analysis and calculated the Absolute Pose Error, defined as APE by Evo Python evaluating package, to evaluate the overall consistency of the out trajectory and ground truth. The absolute pose errors on KITTI sequence 05 and sequence 07 are shown in Figure 9. Figure 9 shows that we achieved global localization with a pose estimation error of not more than 1.69 m and 0.702 m average error, indicating that a low-drift global localization was obtained.

Our proposed SLAM system has a loop-closure-detection module, and thus we verified the accuracy of loop-closure detection on KITTI dataset sequence 00.

**Figure 10:**
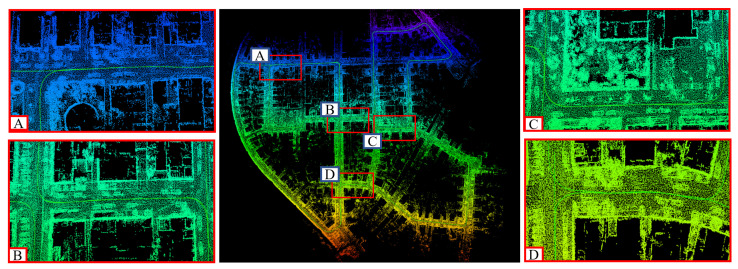
Our mapping result on KITTI dataset sequence 00. (**A**–**D**) are some of the loop closures found in our approach.

As shown in Figure 10, we ensured the global consistency of each loop closure node through pose graph optimization. For example, we focused on the analysis of the road section ABCD, where the vehicle passes twice when collecting data. Intuitively, the point clouds collected by the vehicle passing the same road section twice had no distinct inconsistency on the map. This was precise because of the addition of loop-closure detection, which is running in the background, and thus we realize the consistency of global trajectory, which is also shown in Figure 8a.

**Figure 11:**
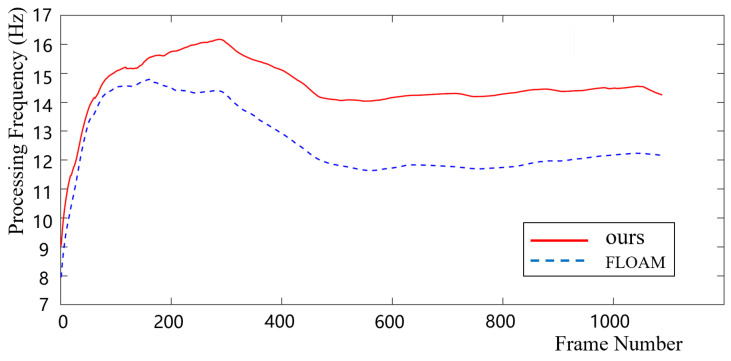
The processing frequency for each pose estimation.

Moreover, in order to verify the real-time performance of our algorithm, we conducted a comparative experiment with our algorithm and FLOAM [13] on the same KITTI dataset, respectively, using a computing platform with not very high computing power, i7-6700HQ mentioned above. Figure 11 shows that the two algorithms can realize real-time pose estimation even using the computing platform with limited resources. On this same platform, we processed 13% faster than FLOAM, which is known for its real-time pose estimation performance. We can conclude that our odometry updates at nearly 15 Hz, faster than the lidar’s 10 Hz. This means that we take full advantage of the lidar data we have collected.

## 4. Discussion

The main goal of our work was to achieve real-time pose estimation on a computing platform with limited resources while minimizing cumulative errors through loop-closure detection and pose graph optimization. Generally, loop-closure detection is always computationally expensive; thus, a SLAM system with loop detection is often difficult to run in real time. Lidar odometry and loop-closure detection are designed to be separated from each other in our SLAM system to solve this problem.

In Section 3, we conducted experiments to verify the mapping performance of our SLAM system. First, the mapping results shown in Figure 7 show excellent global consistency, particularly when compared with the methods without loop-closure detection, such as ALOAM. We also evaluated the absolute error on the *x*-, *y*- and *z*-coordinate, and we can conclude that an unbiased estimation of the positioning in the XY direction was achieved, although the pose estimation in the Z direction is not perfect. Table 1 and Table 2 show the comparison between our method and ALOAM.

The acronyms used in Table 1 and Table 2 represent the average, maximum, median, minimum, root mean squared error, standard deviation and sum of squares for the error of the absolute pose error.

For KITTI sequence 05 with a total length of 2223 m, the average error using our proposed method was only 0.7 m, and the maximum error was no more than 1.7 m. By comparison, since there is no loop-closure detection in ALOAM and FLOAM, the maximum error using ALOAM accumulated to nearly 20 m, while the maximum error using FLOAM accumulated to nearly 10 m. There is no doubt that for the high-precision mapping within 2 km, an error of nearly 20 m is unacceptable.

For the shorter sequence 07 with a total length of 695 m, the average error using our proposed method was only 0.5 m, and the maximum error is 1.1 m, which performed slightly better compared with FLOAM. However, even on the shorter KITTI07, the cumulative error of ALOAM still reached 4 m.

Compared with ALOAM, an important reason for the improvement of the accuracy of our method is loop-closure detection. Therefore, we conduct experiments on the running time of the algorithm to verify the real-time performance of our SLAM system where local lidar odometry and loop-closure detection were designed separated from each other. The experimental results show that the real-time performance of our method was not affected by loop-closure detection and was even faster than FLOAM, which is known for its real-time performance, because of the use of feature submaps for local data association.

In the future, we will attempt to use hierarchical retrieval to accelerate the loop detection process. Different kinds of sensors may be integrated into our framework to further improve the robustness of the method.

## 5. Conclusions

In this paper, a real-time graph-based lidar SLAM system was proposed, which ensured the high accuracy of the local pose estimation and the consistency of the global trajectory. The feature submap-based scan-to-map matching reduced the computational complexity of the data association. In this way, our algorithm can run in real-time even on a device with limited computational resources. The local lidar odometry and loop-closure detection are separated from each other so that the real-time performance of the pose estimation is not affected by loop detection.

The loop detection module running in the background used the submap conducted in the process of real-time pose estimation to describe the local environment and used the scan-to-submap data association similar to the local data association. Therefore, the accuracy of the global data association and the consistency of the global trajectory were guaranteed. Finally, in order to verify the robustness of our method, experiments were conducted on multiple sets of datasets. Experiments showed that, with the addition of the loop-closure-detection module, our method achieved extremely good real-time performance, while the global accuracy was better than other LOAM variants, such as ALOAM and FLOAM.

## Figures and Tables

**Table 1:** The Absolute Pose Error comparison of our method, FLOAM and ALOAM on KITTI05.

Method	Max (m)	Mean (m)	Median (m)	Min (m)	rmse (m)	Std (m)	sse (m${}^{2}$)
ours	1.691626	0.702092	0.627076	0.164140	0.763674	0.300443	1610.2112
FLOAM	9.587101	3.130297	2.464298	0.145527	3.636932	1.851625	36,520.51
ALOAM	19.899526	7.864927	5.898704	0.000000	9.239001	9.239001	192,911.666

**Table 2:** The Absolute Pose Error comparison of our method, FLOAM and ALOAM on KITTI07.

Method	Max (m)	Mean (m)	Median (m)	Min (m)	rmse (m)	Std (m)	sse (m${}^{2}$)
ours	1.084569	0.546476	0.568619	0.080366	0.600000	0.247717	363.5997
FLOAM	1.207170	0.652669	0.640190	0.043527	0.674682	0.170935	501.1696
ALOAM	4.092609	1.652358	1.084602	0.000000	2.055867	1.223233	4437.916
